# Endoscopic Mucosal Resection of a Proximal Esophageal Pyogenic Granuloma

**DOI:** 10.1155/2019/9869274

**Published:** 2019-09-29

**Authors:** Elias Estifan, Varun Patel, Matthew Grossman

**Affiliations:** ^1^Department of Internal Medicine, St. Joseph's University Medical Center, New York Medical College, USA; ^2^Division of Gastroenterology, St. Joseph's University Medical Center, New York Medical College, USA

## Abstract

Pyogenic Granuloma (PG), also known as lobular capillary hemangioma, is usually seen as a polypoid red lesion found on the skin or the mucosal surface of the oral cavity. PG of the gastrointestinal tract is rare, in particular involving the esophagus, only 14 cases have been reported in the English literature. We present an 80-year-old male who underwent endoscopy for evaluation of dysphagia and was found to have a single, red, bilobed 10 mm polyp with adherent white exudate approximately 19 cm from the incisors. Endoscopic ultrasound was performed with a 20 mHz miniprobe which showed the lesion contained to the mucosal layer with no muscularis propria invasion. A decision was made to perform endoscopic mucosal resection (EMR). A mixture of saline and methylene blue was injected into the submucosal plane to raise the lesion with subsequent successful mucosal hot snare resection. The resection defect was then approximated and closed with a hemostatic clip to prevent bleeding. Pathology of the specimen revealed small capillary vessels growing in a lobular architecture with an edematous stroma and a florid inflammatory infiltrate representing a pyogenic granuloma. EMR allows for an en bloc resection of mucosal lesions with tumor-free margins, thereby providing both diagnostic and prognostic information. Comparing EMR with the novel technique of endoscopic submucosal dissection (ESD), the incidence of bleeding and perforation is much lower; making EMR the best and safest resection option for this rare hemangioma. In this case, we demonstrate that EMR is a safe technique in removing a pyogenic granuloma in the esophagus.

## 1. Introduction

Pyogenic Granuloma (PG) is a polypoid form of a hemangioma that frequently occurs on the skin or in the oral cavity. It is a benign tumor with frequent recurrence, with no cases of malignant transformation having been reported [1]. PG of the gastrointestinal tract is rare, in particular involving the esophagus, only 14 cases have been reported in the English literature. Most PG are managed by snare polypectomy, endoscopic mucosal resection (EMR), laser photocoagulation therapy, endoscopic submucosal dissection (ESD) or regional radiation therapy [2]. In our case, we describe the safety of EMR of a pyogenic granuloma in the esophagus.

## 2. Case Report

**Figure 1:**
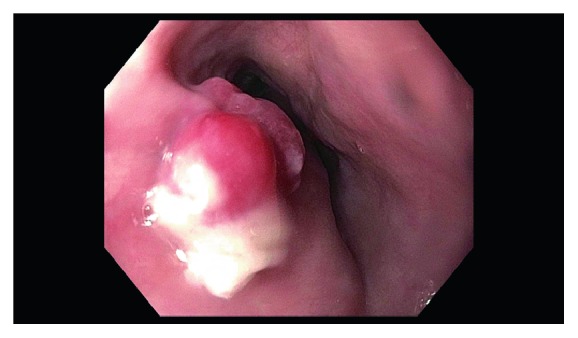
Endoscopic image showing a single, red, bilobed 10 mm polyp with whitish exudate in the proximal esophagus.

**Figure 2:**
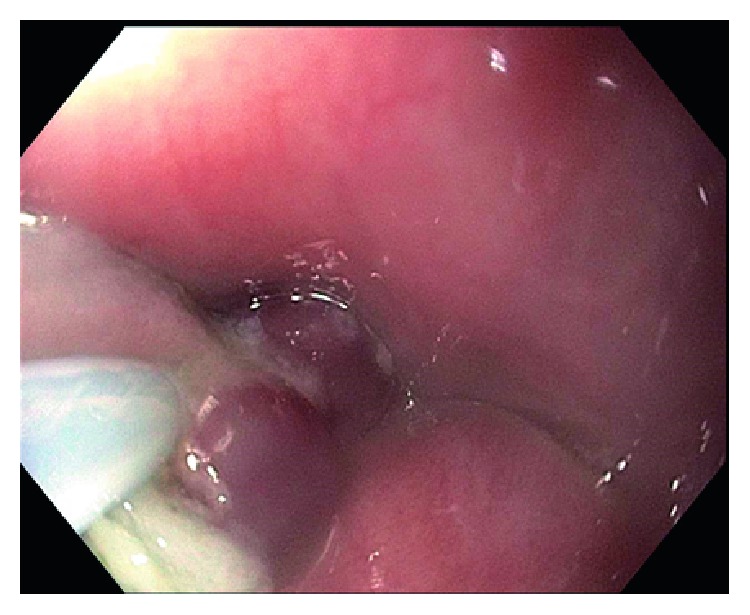
Endoscopic image of the mucosal snare resection being performed after saline and methylene blue injection.

**Figure 3:**
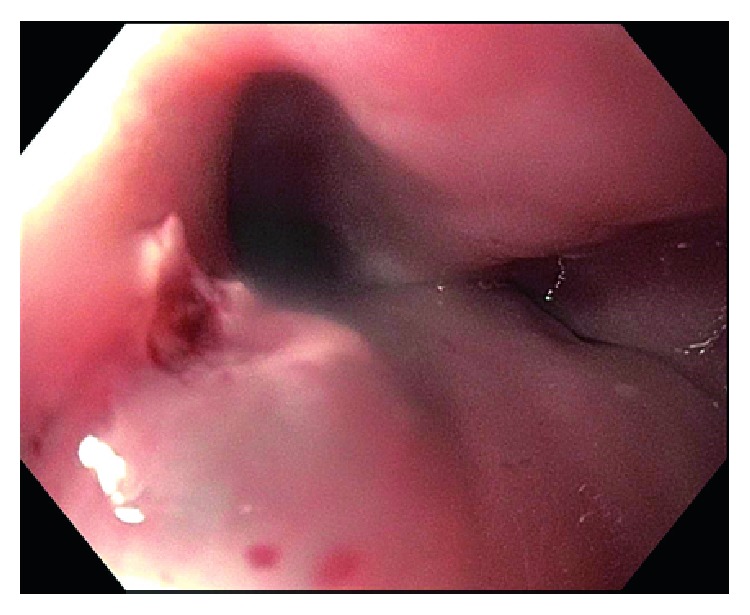
Endoscopic image of the post-polypectomy site with no residual tissue remaining and no evidence of bleeding.

**Figure 4:**
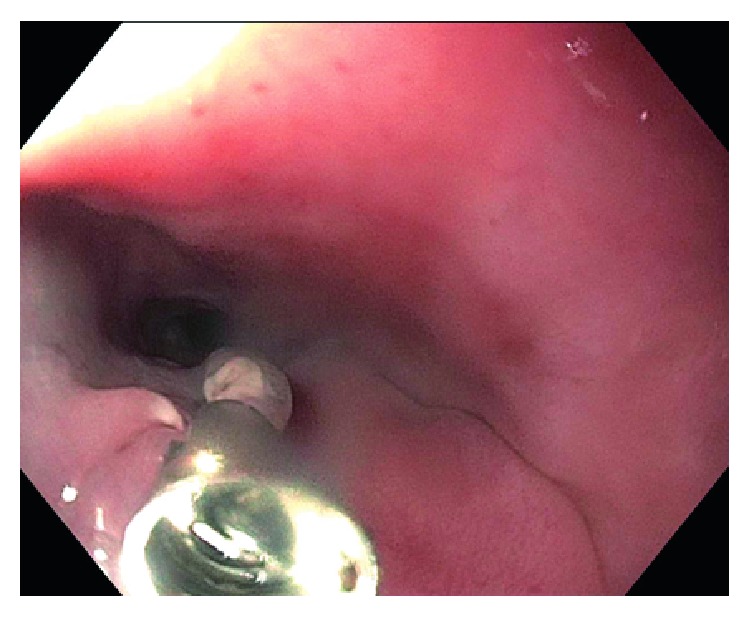
Endoscopic image of successful closure of the post-polypectomy site with a hemostatic clip.

**Figure 5:**
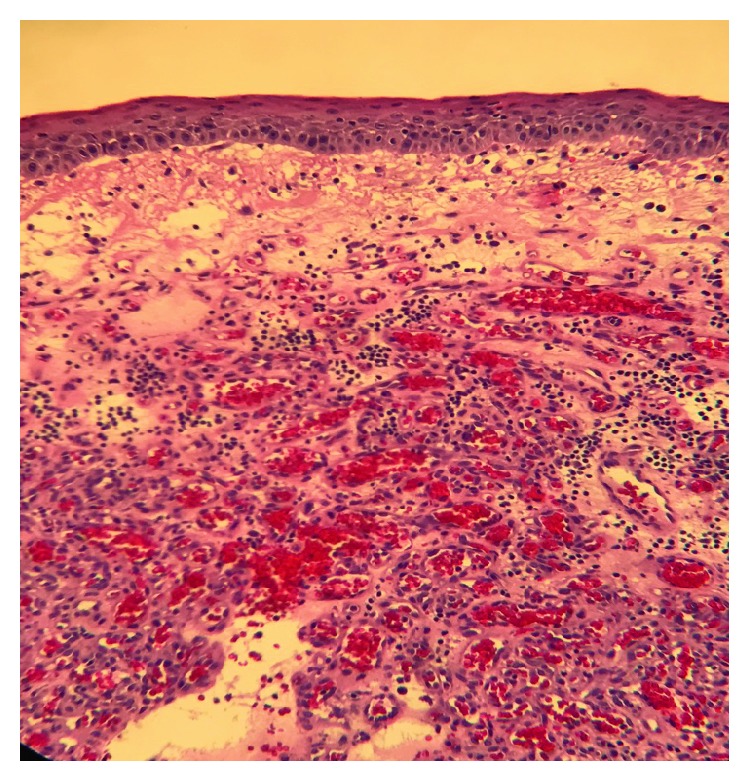
Pathology imaging revealed small capillary vessels growing in a lobular architecture with an edematous stroma and a florid inflammatory infiltrate representing a pyogenic granuloma.

An 80-year-old male with a past medical history of hypertension and diabetes mellitus type II underwent endoscopy for evaluation of dysphagia and was found to have a single, red, bilobed 10 mm polyp with white exudate in the proximal esophagus measuring 19 cm from the incisors (Figure 1). Endoscopic ultrasound was performed with a 20 mHz miniprobe which showed the lesion contained to the mucosal layer with no muscularis propria invasion. A standard gastroscope was introduced and careful examination of the surrounding area and the remaining esophagus was done with no evidence of esophagitis or Barrett's esophagus seen. Thereafter, a mixture of saline and methylene blue was injected into the submucosal plane to raise the lesion. The lesion lifted symmetrically, and a mucosal snare resection was performed successfully with en bloc retrieval of the specimen (Figures 2 and 3). The post-polypectomy site was then carefully examined with no residual tissue or prominent blood vessels seen. The polypectomy defect was then approximated and closed with a hemostatic clip successfully to prevent post-polypectomy bleeding (Figure 4). No bleeding was observed throughout the procedure. Pathology of the specimen revealed small capillary vessels growing in a lobular architecture with an edematous stroma and a florid inflammatory infiltrate representing a lobular capillary hemangioma or pyogenic granuloma (Figure 5).

## 3. Discussion

PG or lobular capillary hemangioma is a hemorrhagic, benign, polypoid neoplasm which was first described in 1897, at that time it was called botryomycosis hominis [3–5]. The lesion is most commonly found on the skin and the mucosal surface of the oral cavity, nasal cavity , and tongue [6, 7]. Only a few cases have been reported in the gastrointestinal tract including the stomach, duodenum, jejunum, ileum, and colon, with the esophagus being the most common [2]. PG is seen in all parts of the esophagus, with the distal segment (57.1%) being most common, followed by the middle segment (35.7%) [2, 6]. On endoscopy, the lesion is usually solitary, appearing in a variety of colors ranging from a bright red/pink to a dark color. Most esophageal PG are confined to the mucosal layer, however submucosal extension has been reported. The size of the lesion can range from several millimeters to a few centimeters. It tends to be friable and hemorrhagic with occasional ulceration and white exudate noted on the surface of the lesion [2, 5, 6, 8, 9].

Most patients who are found to have PG are asymptomatic. However, some symptoms associated with PG are upper gastrointestinal bleeding, epigastric discomfort, chest discomfort, dysphagia, and/or heartburn.

Histological characteristics are proliferation and lobular arrangement of multiple capillaries, inflamed, and edematous stroma, and endothelial cell swelling [1, 2]. The etiology has been a matter of debate as discussed in multiple case reports and its theorized that there is an interplay between mechanical irritation, trauma from foreign body, iatrogenic irritation from biopsy, hormonal factors as in pregnancy, and infections from *Staphylococci*, *Helicobacter pylori*, *Botryomycosis*, *Campylobacter enteritis*, and *Candida* which may affect the morphology of the lesion [5, 10, 11]. Two cases of PG published in the literature were associated with Barrett's esophagus, which illustrates the possibility of PG arising due to chronic gastric acid damage to the esophageal mucosa [1, 7]. In addition, it is important to differentiate the lesion from Kaposi's sarcoma, which typically is encountered in patients with acquired immune deficiency syndrome (AIDS). Kaposi's sarcoma is less well circumscribed, more bluish in color and mostly covered by a moistly glistening intact mucosa. Also, microscopically Kaposi's has slit-like vascular channels lined by atypical endothelial cells which is a feature not found in PG [11]. In our case, there was no identified underlying infection or foreign body irritation that could be linked as an etiologic factor for the formation of a PG.

With the advent of new technology and rapid progression of minimally invasive techniques, endoscopy is becoming integral in providing both diagnostic and therapeutic interventions with virtually no morbidity and mortality [12]. Endoscopy aided with endoscopic ultrasound allows for evaluation of the size, depth, and surface characteristics for early-stage lesions based on Paris classification [13]. EMR, was first described in 1973, is an endoscopic resection technique that provides both diagnostic and prognostic information for mucosal-based lesions [12, 14]. In comparison to cold forceps biopsy, EMR is able to obtain complete tissue resection with a lower recurrence rate [12]. EMR has a high success rate in en bloc resection of mucosal lesions less than 10 mm in size. Lesions that are more extensive up to 20 mm can be resected by EMR; however, ESD has better resection rates with a lower recurrence rates [15]. In one meta-analysis study, the estimate complications of esophageal ESD such as bleeding was 2.1%, whereas the perforation rate was 5.0% [16]. However, in other studies of esophageal EMR, the rate of bleeding post-procedure was between 2.4% and 25% and the perforation rate was equal to ESD of 5.0% [17].

Pyogenic granuloma or lobular capillary hemangioma is a polypoid form of a hemangioma that is usually found as an incidental lesion; however, patients can present with symptoms of dysphagia, bleeding, or pain. Therefore, when discovered on endoscopy, these lesions should be resected to prevent possible complications. EMR provides a lift from the muscularis propria layer to ensure an adequate cushion which minimized the perforation risk, thus entailing a safer resection technique than a polypectomy. We illustrate that EMR, if done appropriately, is the most safe and best treatment modality for such high-risk bleeding lesions.
